# Astrocytic Sonic Hedgehog Alleviates Intracerebral Hemorrhagic Brain Injury *via* Modulation of Blood-Brain Barrier Integrity

**DOI:** 10.3389/fncel.2020.575690

**Published:** 2020-12-03

**Authors:** Gebeili Xing, Tianman Zhao, Xiyue Zhang, He Li, Xiuping Li, Pan Cui, Minshu Li, Daojing Li, Nan Zhang, Wei Jiang

**Affiliations:** ^1^Department of Neurology, Tianjin Neurological Institute, Tianjin Medical University General Hospital, Tianjin, China; ^2^Department of Neurology, Inner Mongolia People’s Hospital, Hohhot, China; ^3^Department of Neurology, Affiliated Hospital of Jining Medical University, Jining, China

**Keywords:** cyclopamine, tight junctions, sonic hedgehog signaling pathway, blood brain barrier, cerebral hemorrhage

## Abstract

**Background**: Intracerebral hemorrhage (ICH) is a fatal subtype of stroke that lacks effective therapy. Blood-brain barrier (BBB) damage is a hallmark of ICH-induced brain injury that leads to edema formation, leukocytes infiltration, influx of blood components into the perihematomal (PHE) region, and eventually brain injury. Astrocytes are essential for the formation and maintenance of the BBB by providing secreted molecules that contribute to the association between these cells. Sonic hedgehog (SHH) derived from astrocytes promotes the maturity and integrity of the BBB by upregulating tight junctions (TJs) in brain capillary endothelial cells (ECs). However, the effect of SHH on BBB in ICH has not been investigated.

**Methods**: Cyclopamine (CYC) is a potent, selective inhibitor that specifically blocks the SHH signaling pathway. Here, we used pharmacological inhibitions (CYC and its derivatives) to determine a critical role of the SHH signaling pathway in promoting BBB integrity after ICH by mechanisms of regulating the TJ proteins *in vivo* and *in vitro*.

**Results**: The expression of astrocytic SHH was upregulated in mouse brains after ICH. Compared with the vehicle-treated group, inhibition of the SHH signaling pathway with CYC and its derivatives treatments aggravated neurological function deficits, brain edema, hematoma volume, and BBB impairment by downregulating TJs in ECs through the SHH-Gli-1 axis *in vivo* and *in vitro*.

**Conclusions**: SHH signaling pathway at the level of the BBB provides a barrier-promoting effect, suggesting that the SHH signaling pathway may function as a potential therapeutic target for restoring BBB function in ICH.

## Introduction

Intracerebral hemorrhage (ICH) is a devastating stroke subtype with a high risk of morbidity and mortality (Xi et al., 2013). It accounts for 10–15% of all stroke but lacks effective treatments. The 1st-year survival rate is only 38% (Dennis et al., 1993; Qureshi et al., 2001). Patients who survive typically have a series of neurologic deficits. Primary brain injury commonly occurs immediately within the first few hours after the ictus of ICH, leading to the direct entry of blood components (thrombin, fibrin, and erythrocyte components) into the brain and forming a hematoma that provokes mechanical damage (Keep et al., 2014; Tschoe et al., 2020). Neurons and glia are affected by the injury and induce oxidative stress, inflammatory activation, neurotransmitter release, membrane depolarization, and mitochondrial dysfunction, contributing to secondary brain injury. Following the activation of coagulation and hemoglobin breakdown, thrombin and clot components were released and initiate the entire cascade of cellular and molecular changes of the brain, leading to the further destruction of brain tissue (Madangarli et al., 2019). After ICH, microglia were rapidly activated to respond to these stimuli by communicating with the immune system and secreting products that cause blood-brain barrier (BBB) destruction, vasogenic edema, neuronal and glial apoptosis (Qureshi et al., 2009; Tschoe et al., 2020). Astrocytes actively accumulate in the perihematomal (PHE) region after ICH onset, participating in modulating BBB function and interaction with microglia and neurotrophic support (Sukumari-Ramesh et al., 2012; Tschoe et al., 2020). Additionally, the integrity of the structure and function of brain microvascular endothelial cells (BMECs) change with ICH progression, and leading to BBB disruption (Yu et al., 2015).

The BBB, which is composed of capillary endothelial cells (ECs), pericytes, and perivascular astrocytes, selectively restricts the blood-to-brain paracellular compound diffusion and regulates cerebral homeostasis and proper neuronal function. After ICH, the increase in leukocyte infiltration into the brain and BBB permeability occurs *via* the changes of paracellular (dysfunction of the tight junction) and transcellular (transcytosis) reactions across the endothelium, the ICH-induced inflammatory response can also cause BBB dysfunction (Keep et al., 2014). Also, Breakdown of the BBB leads to the infiltration of blood components into the brain parenchyma, and the subsequent mechanical stress, the release of toxic components and immune response further aggravate BBB destruction and brain edema.

Astrocytes are important components of the BBB, which not only support the structure of BBB but also regulate BBB integrity *via* secreting factors. Sonic Hedgehog (SHH) is a secreted glycoprotein molecule that participates in various physiological processes, such as neurogenesis, anti-inflammation, anti-oxidation, and anti-apoptosis (He et al., 2017; Caradu et al., 2018; Chen et al., 2018). Recent studies have revealed that SHH produced by astrocytes maintains the tightness and decreases the solute permeability of BBB by upregulating tight junctions (TJs) in ECs (Janzer and Raff, 1987; Alvarez et al., 2011, 2013; Obermeier et al., 2013). BBB ECs express an SHH receptor called patched-1 (Ptch-1), which is a kind of transmembrane protein, suppressed by SHH binding. The G-protein coupled receptor Smoothened (SMO) is activated by the repression of Ptch-1, resulting in the activation of the transcription factor glioma-associated oncogene homolog-1 (Gli-1) and its translocation to SHH-target genes. Gli-1 is an important regulator of the downstream target genes that control the expressions of TJs and BBB formation.

However, the role of the astrocytic SHH signaling pathway in BBB integrity after ICH remains unclear. In this study, we investigated the influence of the astrocytic SHH signaling pathway on ICH and its potential mechanism *in vivo* and *in vitro*.

## Materials and Methods

### Animals, Reagents, and Study Design

All experiments were approved by the Animal Experiments Ethical Committee of Tianjin Neurological Institute and Jining Medical University. Adult male C57BL/6 mice (20–23 g; 7–8 weeks old) were used in this experiment. All mice were housed in an asepsis condition with a controlled temperature of 21°C, a standardized light-dark cycle, and normal amounts of food and water. They were allowed to acclimate for a week before experiments. All operations were performed while the animals were under anesthesia. In our study, we noted no difference in physiologic variables of mice among the experimental groups, including body weight, temperature, blood pressure, and arterial blood gas.

Cyclopamine (CYC; Selleck, Houston, TX, USA) is a small-molecule steroidal alkaloid that specifically inhibits the SHH signaling pathway by blocking SMO and interfering with SHH signal transduction in SHH-responsive cells (Cooper et al., 1998; Chen et al., 2002). *In vivo*, hydroxypropyl-β-cyclodextrin (HPBCD, Sigma–Aldrich, St. Louis, MO, USA) was dissolved in phosphate-buffered saline (PBS; 45% wt/vol) and used to prepare conjugated CYC at 1 mg/ml. Mice were intraperitoneally injected with CYC at a concentration of 10 mg/kg or with the same volume of the vehicle (45% HPBCD in PBS; Palma et al., 2005; Alvarez et al., 2011; Zhang et al., 2017). In cell culture, 10 μM CYC was used to inhibit the SHH signaling pathway (Li et al., 2012; Wang et al., 2014; Sun et al., 2016). LDE-225 (Sonidegib) and GDC-0449 (vismodegib) are two developed drugs targeting the SHH signaling pathway by inhibiting SMO, in a similar way to CYC. The two CYC derivatives are synthetic, highly potent clinical oral medicine approved by the US Food and Drug Administration (FDA) for basal-cell carcinoma (BCC; Irvine et al., 2016; Lauressergues et al., 2016; Rimkus et al., 2016). *In vivo*, carboxymethylcellulose sodium (CMC-NA, Selleck, Houston, TX, USA) was dissolved in double-distilled water (0.5% wt/vol) and used to prepare conjugated LDE-225 at 4 mg/ml and GDC-0449 at 2 mg/ml (Selleck, Houston, TX, USA). Mice were delivered by daily oral gavage with LDE-225 at a concentration of 20 mg/kg and GDC-0449 at a concentration of 10 mg/kg or with the same volume of the vehicle (0.5% CMC-NA in double-distilled water; Pan et al., 2010; Yang et al., 2015; Cazet et al., 2018).

*In vivo*, animals were randomly divided into experimental groups according to the designated treatments: Sham-operated mice group (SHAM), sham-operated mice treated with CYC/ LDE-225/ GDC-0449 group (SHAM+CYC/SHAM+LDE-225/SHAM+GDC-0449), ICH-operated mice treated with the vehicle group (ICH+Vehicle), ICH-operated mice treated with CYC/ LDE-225/ GDC-0449 group (ICH+CYC/ICH+LDE-225/ICH+GDC-0449). In groups treated with vehicle and CYC, mice received a daily injection of an equal volume of vehicle or CYC for three consecutive days starting from the day of operation (Figure 2A). In groups treated with vehicle and LDE-225/ GDC-0449, mice received daily oral gavage of an equal volume of vehicle or LDE-225/ GDC-0449 for three consecutive days starting from the day of operation (Figures 3A,E). *In vitro*, cells were assigned into four groups: ECs cultured alone (bEnd3), ECs co-cultured with sham-astrocytes (SHAM), ECs co-cultured with ICH-astrocytes and vehicle (ICH+Vehicle), ECs co-cultured with ICH-astrocytes and CYC (ICH+CYC).

**Figure 1:**
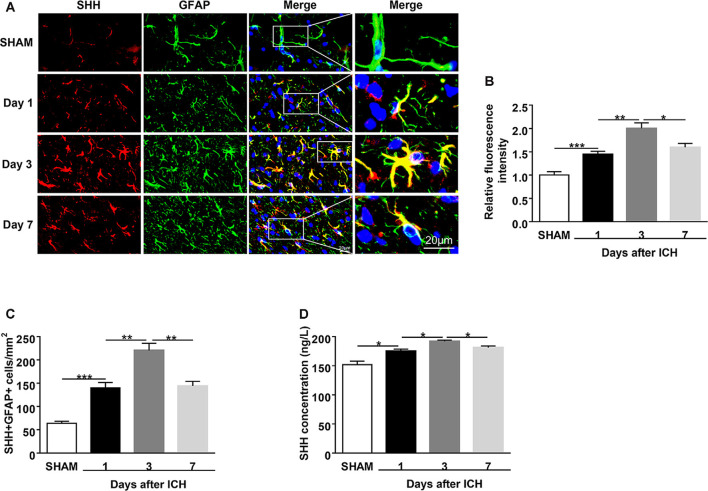
Upregulation of sonic hedgehog (SHH) expression in the astrocytes of intracerebral hemorrhage (ICH) mice. ICH was induced in mice by injection of autologous blood.** (A,B)** Representative images and quantification of fluorescence intensity showed SHH (red) in astrocytes (glial fibrillary acidic protein, GFAP, green) in mouse perihematomal (PHE) brain tissues at days 1, 3, and 7 after sham or ICH surgery, *n* = 6 mice per group. **(C)** Quantification of the SHH^+^GFAP^+^ cells in mouse PHE brain tissues at days 1, 3, and 7 after surgery, *n* = 6 mice per group. **(D)** The protein levels of SHH in mouse PHE brain tissues at days 1, 3, and 7 after sham or ICH surgery were measured by ELISA kits, *n* = 3 mice per group. Data are presented as mean ± SEM. *P < 0.05, **P < 0.01, ***P < 0.001.

**Figure 2:**
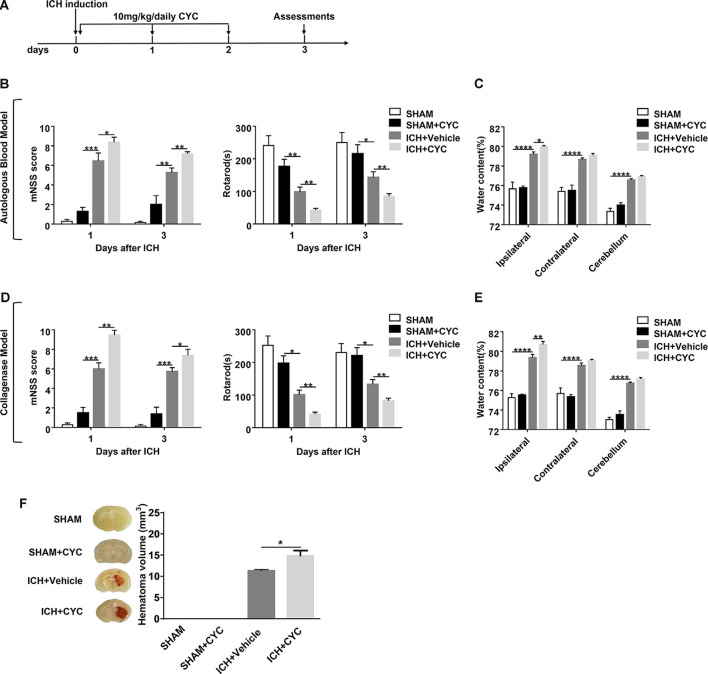
Blockage of the SHH signaling pathway by cyclopamine (CYC) aggravates ICH-induced brain injury in mice. **(A)** The schematic diagram illustrates the CYC treatment and study design. Mice were treated with CYC (10 mg/kg) or an equal volume of vehicle by intraperitoneal injection once a day, starting from day 0 to day 2 after sham or ICH operation. **(B,D)** The neurological function assessments of modified Neurological Severity Score (mNSS) and rotarod tests were performed to evaluate the motor, sensory, reflex, and balance functions in mice treated with CYC or vehicle on day 1 and day 3 after injection of saline, autologous blood **(B)**, or collagenase **(D)**, *n* = 6–11 mice per group. **(C,E)** Measurement of brain water content in mice treated with CYC or vehicle at day 3 after injection of saline, autologous blood **(C)**, or collagenase **(E)**, *n* = 3–8 mice per group. **(F)** Measurement of hematoma volume in mice treated with CYC or vehicle at day 3 after injection of saline or collagenase, *n* = 3 mice per group. Data are presented as mean ± SEM. *P < 0.05, **P < 0.01, ***P < 0.001, ****P < 0.0001.

**Figure 3:**
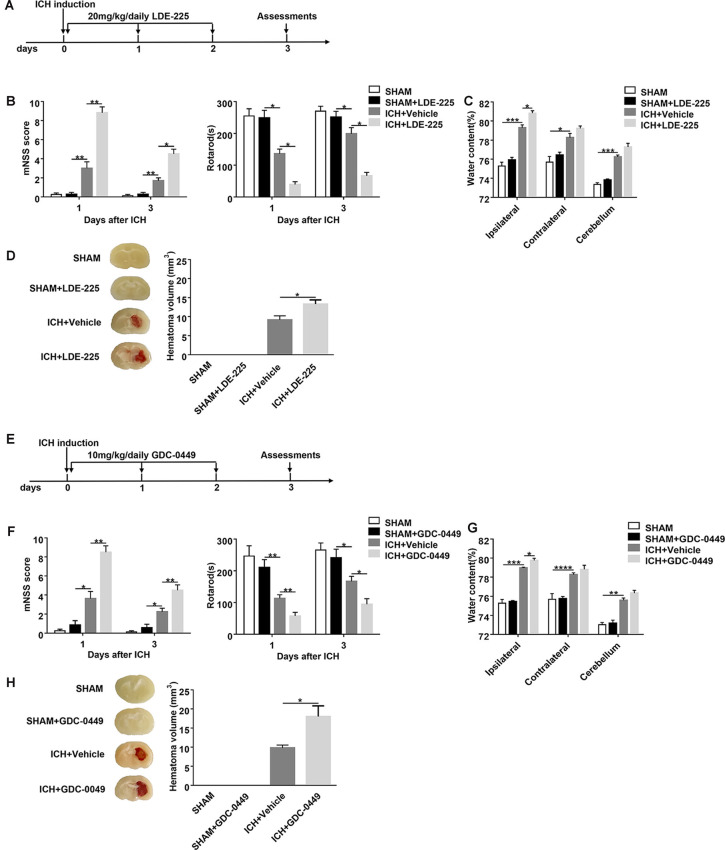
Blockage of SHH signaling pathway by CYC derivatives (LDE-225 and GDC-0449) aggravates ICH-induced brain injury in mice. **(A,E)** The schematic diagram illustrates LDE-225 or GDC-0449 treatment and study design. Mice received daily oral gavage of LDE-225 (20 mg/kg) or GDC-0449 (10 mg/kg), or with an equal volume of vehicle, starting from day 0 to day 2 immediately after sham or ICH operation. **(B,F)** The neurological function assessments of mNSS and rotarod tests were performed to evaluate the motor, sensory, reflex, and balance functions in mice treated with LDE-225/GDC-0449 or vehicle on day 1 and day 3 after injection of saline or collagenase, *n* = 6–10 mice per group. **(C,G)** Measurement of brain water content in mice treated with LDE-225/GDC-0449 or vehicle on day 3 after injection of saline or collagenase, *n* = 3 mice per group. **(D,H)** Measurement of hematoma volume in mice treated with LDE-225/GDC-0449 or vehicle on day 3 after injection of saline or collagenase, *n* = 3 mice per group. Data are presented as mean ± SEM. *P < 0.05, **P < 0.01, ***P < 0.001, ****P < 0.0001.

### Establishment of Mouse ICH Model

In this study, to confirm our findings in ICH, an autologous blood-induced ICH model was also involved to exclude the potential influence of CYC on collagenase. Injection of collagenase or autologous blood was used to induce ICH in mice as previously described (Grossetete and Rosenberg, 2008; Rynkowski et al., 2008; Li et al., 2017a,c). A mixture of ketamine (100 mg/kg) and xylazine (10 mg/kg) was used to anesthetize mice by intraperitoneal injection. On the right side of the mouse skull, a 1 mm burr hole was drilled (stereotaxic coordinates: 0.5 mm anterior to the Bregma and 2.3 mm lateral from the midline). The collagenase injection model was induced: 0.0375 U bacterial collagenase (Type IV; Solarbio, Beijing, China) in 0.5 μl saline was injected at a rate of 1 μl/min into the right caudate nucleus (3.7 mm below the skull surface) using a 10 μl syringe driven by an infusion pump (KD Scientific, Holliston, MA, USA). The autologous blood injection model was induced 30 μl autologous blood was obtained from the angular vein of the mouse and then injected using a 50 μl syringe driven by an infusion pump (KD Scientific, Holliston, MA, USA) as previously described (Li et al., 2017c). The first 5 μl autologous blood was infused at a depth of 3 mm with a rate of 1 μl/min to generate a clot under the hole. The needle was then placed to a depth of 3.7 mm and paused for 5 min. After the pause, the remaining 25 μl of autologous blood was infused at the same rate. Then the needle was slowly withdrawn at 1 mm increments after pausing for 20 min, each for three movements at the 5-min interval. In the sham group, mice were injected with the same dose of saline. Ultimately, the skull hole was sealed with sterile bone wax and the skin incision was sterilized and sutured using standard techniques. Then animals were placed in standard mouse cages with a free supply of water and food.

### Neurological Behavioral Assessment

Neurological behavioral assessment including the modified Neurological Severity Score (mNSS; Jin et al., 2017; Li et al., 2017) and the Rotarod test (Moriguchi et al., 2012; Yang et al., 2018) was performed on days 1 and 3 after sham and ICH surgery by two observers who were blinded to the experiments.

The mNSS test is a composite measurement to comprehensively assess the motor (test of raising mouse by the tail and placing the mouse on the floor), sensory (visual test, tactile test, and proprioceptive test), reflex (reflexes absent and abnormal movements) and balance (beam balance tests) functions (Supplementary Table 1; Li et al., 2017b). The scale of the mNSS is from 0 to 18. A score of 13–18 indicates severe impairment, 7–12 indicates moderate impairment, and 1–6 indicates mild impairment (Ren et al., 2018). One point was given if mice failed to complete a task or lacked a tested reflex.

The rotarod test measures the systemic motor abilities, specifically the balance and coordination of the ICH mice (Yang et al., 2018). Mice practiced running on an automated accelerating rotarod treadmill (3 cm in diameter and 30 cm length, with a non-slippery surface 20 cm above the base). The rod contained five equal sections, which were divided by four disks, enabling five mice to walk on the rod at the same time. Test sessions consisted of three trials, in which the fixed acceleration mode’s rotational speed accelerated from 0 to 40 rpm. Each trial lasted for 5 min with a 30-min interval between two trials. The final score was recorded as the average time over three trials while mice were able to remain on the rotarod. Mice were positioned on the rotating rod drum up to 5 min. Before ICH, mice were tested on the rotating rod (4 rpm) over 3 days, and only those who stayed on the rod for at least 60 s were subjected to ICH.

### Measurement of Brain Water Content

Brain water content was assessed at day 3 after sham and ICH surgery (Han et al., 2017). Mice brains were harvested and separated into three parts without perfusion: the left cerebral hemisphere, the right cerebral hemisphere, and the cerebellum. The samples were quickly weighed on a precise electronic scale (wet weight) and then dried at 100°C for 24 h to measure the dry weight. The following formula was used to calculate the percentage of edema in the mouse brain: (wet weight − dry weight)/wet weight × 100%.

### Assessment of Hemorrhage Volume

On day 3 after sham and ICH surgery, mouse brains were collected and post-fixed in 4% paraformaldehyde overnight at 4°C. In slices of 1 mm thickness, hemorrhage sizes were gaged. After photographing these slices, hematoma volumes were measured using ImageJ software (NIH, Bethesda, MD, USA, 1997–2009^1^).

### Immunofluorescent Staining and Analysis

On day 3 after sham and ICH surgery, mouse brains were collected and post-fixed in 4% paraformaldehyde overnight at 4°C. The fixed brains were then removed and dehydrated using 15% and 30% sucrose in PBS. Subsequently, brain sections (8 μm) were prepared using a Leica Microsystems LM3050S cryostat (Leica Microsystems, Wetzlar, Hessen, Germany). Brain tissues were permeabilized with 0.3% Triton X-100 for 10 min. After blocking with 3% bovine serum albumin for 1 h at ambient temperature, slides were incubated with primary antibodies: rat anti-SHH (25 μg/ml; Abcam, Cambridge, MA, USA), goat anti-glial fibrillary acidic protein (GFAP; 1:500; Abcam, Cambridge, MA, USA), rabbit anti-Ptch-1 (1:500; Affinity Biosciences, Cincinnati, OH, USA), mouse anti-SMO (1:100; Santa Cruz Biotechnology, Santa Cruz, CA, USA) and mouse anti-Gli-1 (1:100; Santa Cruz Biotechnology, Santa Cruz, CA, USA) overnight at 4°C. For *in vitro* staining, cells were inoculated on coverslips, fixed, and incubated with the following primary antibodies: rabbit anti-claudin-5 (1:100; Abcam, Cambridge, MA, USA) and rabbit anti-ZO-1 (1:100; Abcam, Cambridge, MA, USA). The next day, cells were washed with PBS (5 × 5 min) and then incubated at ambient temperature for 1 h with designated secondary antibodies (1:1,000; all from Invitrogen, Carlsbad, CA, USA): rabbit anti-rat 594, rabbit anti-goat 488, goat anti-mouse 488, donkey anti-rabbit 488. Finally, tissues were counterstained for cell nuclei with 4′,6-diamidino-2-phenylindole. The intensity of the staining was measured semi-quantitatively using ImageJ software. The cell numbers were expressed as the SHH+ GFAP+ cell numbers per square millimeter.

### ELISA

We separately collected samples of mice brain at 1, 3, and 7 days after ICH and the co-cultured supernatants for 24 h to determine the contents of SHH and MMP-9 by ELISA assay (SenBeiJia, Nanjing, China), according to the manufacturer’s protocol under uniform conditions. Determinations were performed in duplicate on the individual sample. Results expressed from each sample were normalized for protein concentration.

### Measurement of Evans Blue

The BBB integrity was evaluated using 2% Evans Blue dye (EB; 2 ml/kg; Solarbio, Beijing, China), which was injected into the tail vein 2 h before sacrifice at day 3 after sham and ICH surgery. Mouse brain tissues were harvested and the ipsilateral hemisphere was weighed on an electronic balance. After homogenization with formamide in a test tube, the ipsilateral hemisphere was incubated at 60°C for 72 h. After centrifuged at 1,000× rpm for 5 min, the supernatants were collected and added into a microplate along with standards. The optical density of the extracted dye in each sample was assessed using a microplate reader (Thermo Scientific, San Diego, CA, USA; *λ* = 450, 570 nm). The following formula was used to calculate the concentration of EB by linear regression: EB content in brain tissue (μg/g wet brain) = EB concentration × formamide (ml)/wet weight (g).

### Western Blot Analysis

On day 3 after sham and ICH surgery, the fresh ipsilateral hemispheres of mouse brains samples were homogenized by sonication in RIPA buffer (Solarbio, Beijing, China) containing protease complete inhibitor cocktail. The supernatants were collected after centrifugation. *In vitro*, after co-cultured for 24 h, cells were harvested into RIPA buffer containing cocktail. The Protein concentration was assessed by using the BCA protein assay kit (Solarbio, Beijing, China). Protein samples were separated on 10% SDS-PAGE gels and then transferred onto PVDF membranes (Millipore, Billerica, CA, USA), which were blocked with 5% non-fat milk for 1 h at ambient temperature. The membranes were incubated at 4°C overnight with the following primary antibodies: rabbit anti-ZO-1 (1:1,000; Millipore, Billerica, CA, USA), rabbit anti-Occludin (1:1,000; Millipore, Billerica, CA, USA), rabbit anti-Claudin-5 (1:1,000; Millipore, Billerica, CA, USA), mouse anti-SMO (1:1,000; Santa Cruz Biotechnology, Santa Cruz, CA, USA), mouse anti-Gli-1 (1:1,000; Santa Cruz Biotechnology, Santa Cruz, CA, USA) and mouse anti-glyceraldehyde 3-phosphate dehydrogenase (GAPDH; 1:1,000; ZSGB-BIO, Beijing, China). The next day, the membranes were washed and incubated with a species-appropriate secondary antibody for 1 h at ambient temperature. After three washes, the intensity of the protein band was examined by densitometry (Bio-Rad, Hercules, CA, USA) which was analyzed using ImageJ software.

### Isolation of Primary Astrocytes

Primary astrocytes were sorted from mouse brain tissues at day 3 after sham and ICH surgery as previously described (Li et al., 2018; Scheuer et al., 2019). Briefly, brains were gently removed, chopped with scissors in ice-cold Dulbecco’s Modified Eagle’s Medium, and then digested with papain (Sigma–Aldrich, St. Louis, MO, USA) at 37°C for 30 min. Then cell suspensions were fractionated by 30% Percoll gradients at 700 × g for 10 min to get rid of myelin. After resuspended in 1% fetal bovine serum, cell suspensions were filtered with a cell strainer to obtain mononuclear cells. For magnetic labeling of astrocytes, 1 × 10^6^ cells per 60 μl 1% fetal bovine serum were incubated with anti-Glast-1 antibody, which is marked with PE (Novus, Saint Charles, Missouri, MO, USA) for 30 min at 4°C. After washed with buffer, 1 × 10^6^ cells per 20 μl buffer were incubated with anti-PE microbeads (Miltenyi Biotec, Bergisch Gladbach, NRW, Germany) for 20 min at 4°C. Magnetically labeled astrocytes were then isolated after several washes with 2 ml buffer. The purity of the primary astrocytes was determined by flow cytometry (BD Biosciences, San Jose, CA, USA) before culture (>98%).

### Cell Culture and Treatment

A mouse brain endothelial cell line, bEnd3 (Zqxzbio, Shanghai, China), was used in this study. bEnd3 cells were seeded into 24-well plates with a density of 1 × 10^5^ cells per well and grown in high-glucose Dulbecco’s Modified Eagle’s Medium. After culturing the bEnd3 cells in a 5% CO_2_/95% air incubator at 37°C for 3 days, primary astrocytes were seeded into the plate with a density of 1 × 10^5^ cells per well and co-cultured with the bEend3 cells for 24 h. Then, co-cultured cells were incubated with CYC or an equal volume of the vehicle for 24 h before harvesting for subsequent treatments.

### Statistical Analysis

Data were shown as mean ± SEM and analyzed using GraphPad Prism 6.0 software (GraphPad Software, La Jolla, CA, USA). Two-tailed unpaired student’s *t*-test was used to determine the differences between the two groups. One-way ANOVA (Kruskal-Wallis test) followed by the Tukey *post hoc* test or 2-way ANOVA followed by the Bonferroni *post hoc* test was used for multigroup comparison. *P* < 0.05 was considered statistically significant.

## Results

### Upregulation of SHH Expression in Astrocytes of the Perihematomal (PHE) Brain Tissues After ICH

To identify the brain expression of SHH after ICH, we injected mice with autologous blood and measured the expression of SHH in the brain using immunostaining and ELISA at days 1, 3, and 7 after ICH. We found that SHH was mainly expressed in astrocytes and its expression was significantly increased on the first day after ICH as compared to the sham group, the protein level of SHH was persistently increased on day 3, but downregulated at day 7 after ICH (Figures 1A–D). The dynamic expression of SHH in the brain indicates the participation of astrocytic SHH in the pathogenesis after ICH.

### Inhibition of SHH Signaling Pathway Aggravates Neurological Deficits and Brain Injury in Mice After ICH

To assess the effect of the SHH signaling pathway on ICH, we used SMO inhibitors (CYC, LDE-225, and GDC-0449), antagonists of SHH downstream key molecule SMO, to selectively inhibit the activation of the SHH signaling pathway. There was no significant change in neurological deficits and brain injury in SMO inhibitors-treated mice. After ICH surgery, neurological deficits and brain injury were aggravated. We found that mice treated with SMO inhibitors showed severer neurological function deficits compared with the ones receiving vehicle at day 1 and day 3 after ICH surgery (Figures 2B,D, 3B,F). Compared with the vehicle-treated group, the brain water contents in mice injected with SMO inhibitors were significantly increased at day 3 after ICH surgery (Figures 2C,E, 3C,G). Compared with the vehicle-treated group, the hematoma sizes in mice injected with SMO inhibitors were significantly increased at day 3 after ICH surgery (Figures 2F, 3D,H). Meanwhile, hematoma sizes showed to be correlated with the behavioral scores in CYC-treated mice. Severer neurological deficits tend to be found in mice with larger hematoma sizes (Supplementary Figure 2). These data suggest that the inhibition of the SHH signaling pathway exacerbates ICH-induced brain injury.

### Blockage of SHH Signaling Pathway Exacerbates BBB Damage in Mice After ICH

**Figure 4:**
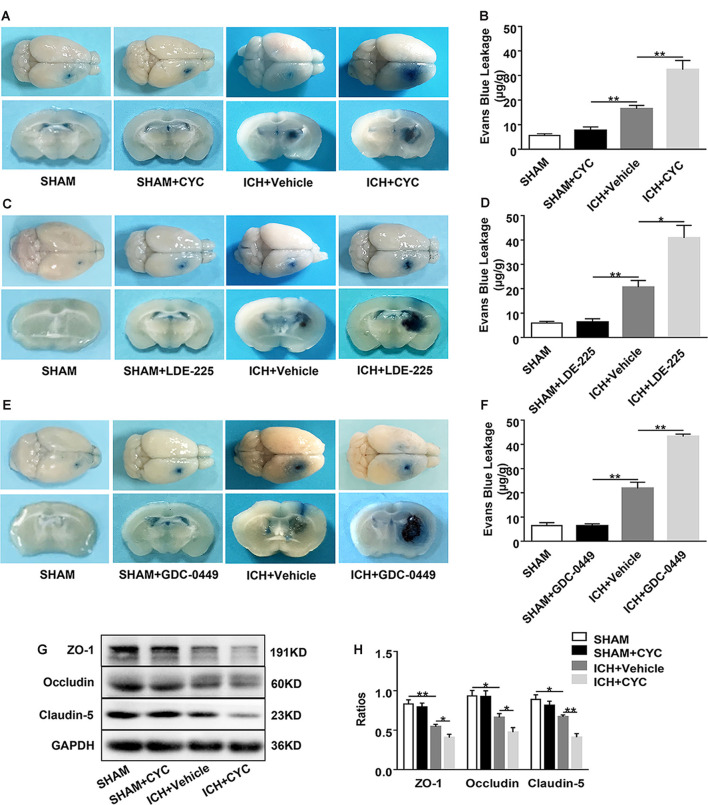
Inhibition of the SHH signaling pathway aggravates blood-brain barrier (BBB) permeability in mice after ICH. ICH was induced in mice by injection of collagenase. Mice received intraperitoneal injections of CYC (10 mg/kg) or an equal volume of the vehicle once a day, starting from day 0 to day 2 immediately after sham or ICH operation. Mice received daily oral gavage of LDE-225 (20 mg/kg) or GDC-0449 (10 mg/kg), or with an equal volume of vehicle, starting from day 0 to day 2 immediately after sham or ICH operation. The BBB integrity of mouse brain tissues was assessed at day 3 after sham or ICH surgery. **(A–F)** Histology images showed the Evans Blue (EB) dye leakage in the brain tissues of mice treated with SMO inhibitors or vehicle, *n* = 3–7 mice per group. **(G,H)** Western blot images showed the expressions of tight junctions (TJs) in the brain tissues of mice treated with CYC or vehicle, *n* = 4–8 mice per group. Data are presented as mean ± SEM. *P < 0.05, **P < 0.01.

To identify the role of SMO inhibitors in BBB dysfunction after ICH, EB extravasation, and the expressions of TJs were examined. Compared with the sham group, there was no significant change of EB extravasation in SMO inhibitors-treated mice. After ICH surgery, EB extravasation was increased. Compared with the vehicle-treated group, SMO inhibitors treatments increased the leakage of BBB at day 3 after ICH (Figures 4A–F). Western blot analysis of TJs also showed the decrease of brain expressions of ZO-1, occludin, and claudin-5 in ICH+CYC groups at day 3 after ICH surgery as compared to the vehicle-treated controls (Figures 4G,H). These data indicate that the SHH signaling pathway has a critical effect on BBB integrity after ICH.

### Inhibition of SHH Signaling Pathway Downregulates the Expressions of Tight Junctions in Endothelial Cells After ICH *in vitro*

**Figure 5:**
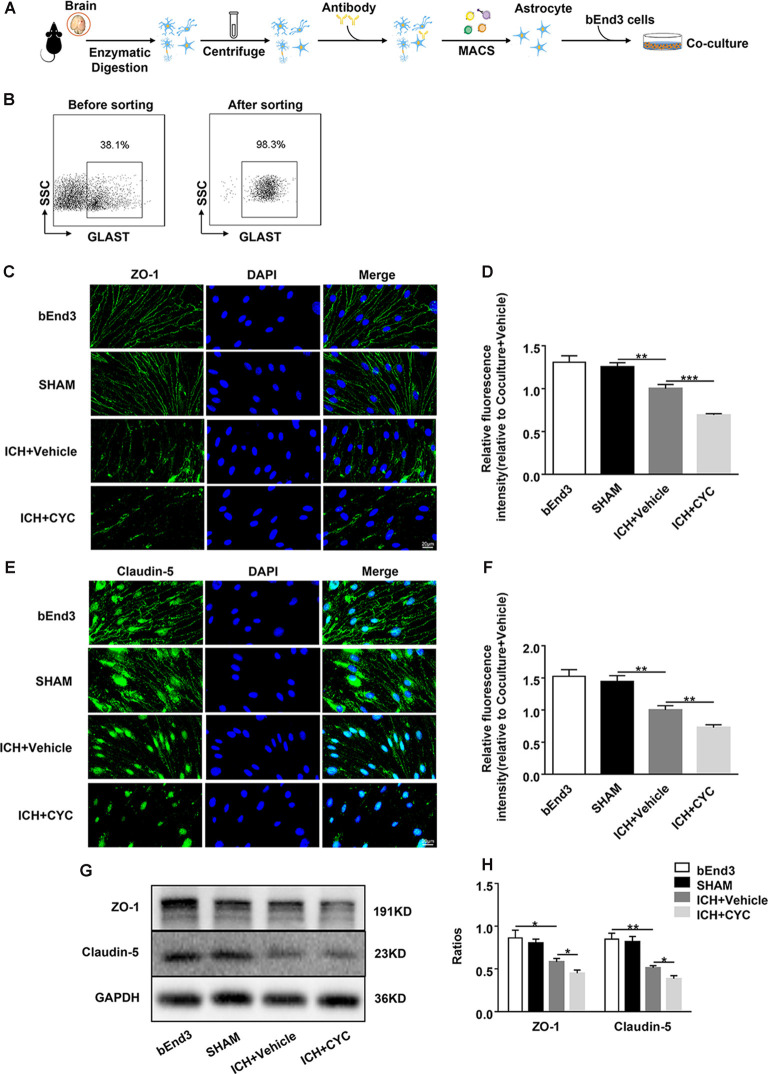
Astrocytic SHH signaling pathway regulates the expressions of TJs in astrocyte-endothelial co-culture *in vitro*. ICH was induced in mice by injection of autologous blood. **(A,B)** Procedures for the isolation and purification of astrocytes (GLAST+) from sham and ICH mice brains. On day 3 after surgery, brain tissues of mice were harvested. **(C–F)** Representative images and quantification of fluorescence intensity showed the expressions of ZO-1 and claudin-5 in bEnd3 cells that were cultured alone, co-cultured with sham-induced astrocytes, co-cultured with ICH-induced astrocytes and vehicle, and co-cultured with ICH-induced astrocytes and CYC. *n* = 6 mice per group. **(G,H)** Western blot images showed the expressions of ZO-1 and claudin-5 in bEnd3 cells that were cultured alone, co-cultured with sham-induced astrocytes, co-cultured with ICH-induced astrocytes and vehicle, and co-cultured with ICH-induced astrocytes and CYC. *n* = 6 mice per group. Data are presented as mean ± SEM. *P < 0.05, **P < 0.01, ***P < 0.001.

To further explore whether the SHH signaling pathway is involved in the regulation of TJs after ICH, we co-cultured astrocytes with brain ECs. Astrocytes were sorted from the brain tissues of sham and ICH mice on day 3 after surgery. They were co-cultured with bEend3 cells for 24 h (Figures 5A,B) followed by the treatment with CYC or vehicle. To explore the effects of astrocytic SHH on TJs, we measured the expressions of ZO-1 and claudin-5 in bEend3 cells. There were no obvious differences between ZO-1 and claudin-5 expressions between the bEnd3 group and the SHAM group. Compared with the two groups above, the ICH+Vehicle group showed a significant reduction in the expressions of ZO-1 and claudin-5 in bEnd3 cells. Furthermore, the ICH+CYC group showed a robust reduction in the expressions of ZO-1 and claudin-5 in bEnd3 cells as compared with the vehicle-treated group (Figures 5C–H). These data clarify the vital role of the astrocytic SHH signaling pathway in promoting BBB integrity *via* the regulation of TJs expressions.

### Blockage of SHH Signaling Pathway Suppresses ICH-Induced Activation of SHH-Gli-1 Axis in ICH Mice

**Figure 6:**
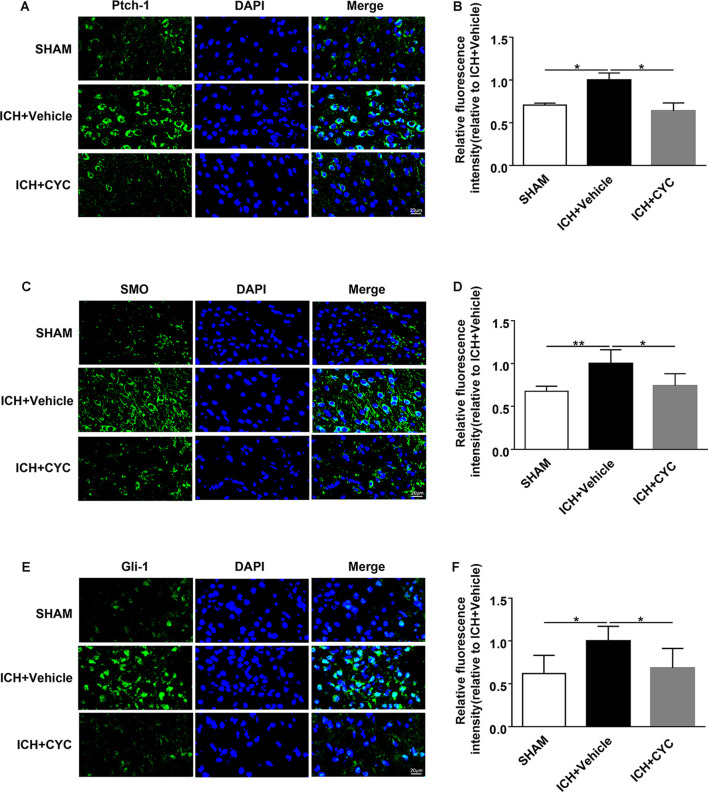
CYC inhibits the activation of the SHH-Gli-1 axis in ICH mice. ICH was induced in mice by injection of collagenase. Mice received CYC (10 mg/kg) or an equal volume of the vehicle *via* intraperitoneal injection once a day, starting from day 0 to day 2 after sham or ICH operations. On day 3, brain samples were prepared and the expressions of the downstream molecules of the SHH-Gli-1 axis were measured by immunostaining. **(A,B)** Representative images and quantification of fluorescence intensity showed the expressions of Ptch-1 in mice received CYC treatment or vehicle on day 3 after ICH, *n* = 3 mice per group.** (C,D)** Representative images and quantification of fluorescence intensity showed the expressions of SMO in mice received CYC treatment or vehicle on day 3 after ICH, *n* = 5 mice per group. **(E,F)** Representative images and quantification of fluorescence intensity showed the expressions of Gli-1 in mice received CYC treatment or vehicle on day 3 after ICH, *n* = 5 mice per group. Data are presented as mean ± SEM. *P < 0.05, **P < 0.01.

Based on the above results, we performed further experiments to identify the downstream mechanism under CYC inhibition of the SHH signaling pathway. The expressions of SHH-Gli-1 axis-related proteins were examined using immunofluorescent staining. Results showed that the expressions of Ptch-1, SMO, and Gli-1 in ICH+Vehicle groups were increased as compared to the SHAM group, indicating the SHH signaling pathway activation induced by ICH. The expressions of Ptch-1, SMO, and Gli-1 in ICH+CYC groups were decreased compared to ICH+Vehicle groups on day 3 after ICH surgery (Figures 6A–F). To further elucidate the SHH pathways under CYC inhibition, changes of SMO and Gli-1 were also identified *in vitro* (Supplementary Figure 3). These findings suggest that the inhibition of the SHH signaling pathway by CYC treatment suppresses the activation of the SHH-Gli-1 axis in mice with ICH.

## Discussion

**Figure 7:**
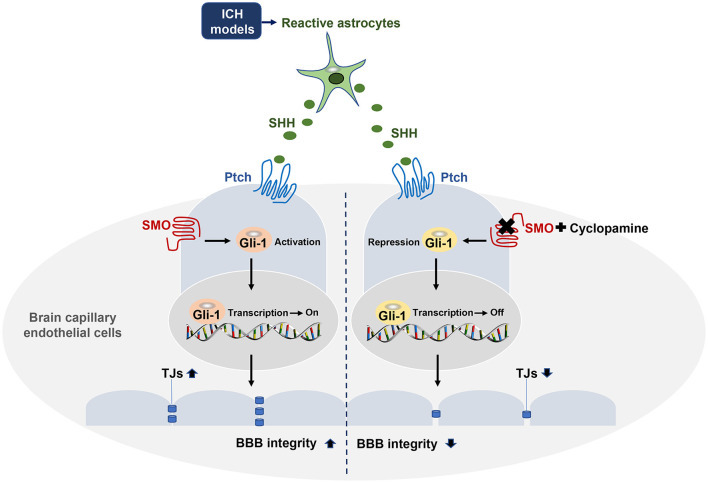
Astrocytic SHH signaling pathway regulates BBB integrity after ICH. SHH secreted by astrocytes binds to its receptor Ptch-1 in endothelial cells (ECs), leading to the activation of SMO and its downstream transcription factor Gli-1. Activated Gli-1 upregulates TJs and enhances BBB integrity. Upon the binding of SMO to CYC, this pathway is repressed, resulting in decreased TJs expressions and exacerbated BBB permeability.

This study provides novel evidence that astrocytic SHH plays a protective role in BBB integrity after ICH. We demonstrated that the level of astrocytic SHH was significantly elevated in PHE brain tissue of the animal model in the early stage of ICH, and astrocytes were the primary cell type that expressed SHH. The inhibition of the SHH signaling pathway using SMO inhibitors treatment aggravated neurological impairments, brain edema, hematoma volume, and BBB permeability in the early stage of ICH in mice. Furthermore, the *in vivo* and *in vitro* data proved that astrocytic SHH improved the expressions of TJs in ECs through the SHH-Gli-1 axis and thus played a protective role in BBB integrity (Figure 7). Taken together, these results indicate that astrocytic SHH may be a potential therapeutic target for the management of ICH injury.

Astrocytes are essential for the maintenance of BBB structural integrity. They connect to the CNS vasculature and their end-feet sheathe the brain microvessels (Sukumari-Ramesh et al., 2012). Several studies suggested that a range of astrocyte-derived factors are responsible for BBB repair after acute brain injury (Michinaga and Koyama, 2019). After ICH, activated astrocytes are involved in the development of cytotoxic edema *via* expressing aquaporin-4, a water channel protein released from the end-feet of astrocytes (Laird et al., 2010). The role of astrocytes is known to be crucial to the SHH signaling pathway as astrocytes are considered a primary cellular source of SHH production in CNS injury (Amankulor et al., 2009; Alvarez et al., 2011; He et al., 2013; Bohannon et al., 2019). The upregulation of SHH in astrocytes was reported in TBI, EAE, and LPS injury models (Wang et al., 2008; Amankulor et al., 2009). One recent research demonstrated that CNS injuries induce a high SHH expression in mice brains, and astrocytes can act like stem cells with neurosphere formation *in vitro* (Sirko et al., 2013). Here, we firstly clarified the protective effect of astrocyte-derived SHH on BBB integrity after ICH. These data supported the notion that astrocytes contributed to the formation and progression of ICH-induced brain edema.

Many previous studies have used co-culture with primary astrocytes or astrocyte-conditioned media to enhances BBB characteristics and formation of TJs in brain ECs (Arthur et al., 1987; Isobe et al., 1996; Gaillard et al., 2001). However, some evidence indicates that co-culture with astrocytes or astrocyte-conditioned media didn’t change the expressions of TJs in brain ECs (Brown et al., 2007; Li et al., 2010). The possible reasons for the opposite results may attribute to the variances in the culture models, culture conditions, and cell lines. Thus, the factors produced in the co-cultured environment may affect the BBB permeability. Astrocyte-derived endothelins (ETs), nitric oxide (NO), and vascular endothelial growth factors (VEGFs) cause reduction of TJs and endothelial apoptosis in BBB dysfunction, on the contrary, apolipoprotein E (APOE), insulin-like grow factor-1 (IGF-1), glial-derived neurotrophic factor (GDNF) and retinoic acid (RA) cause increase of TJs and protect ECs, resulting in BBB repair (Michinaga and Koyama, 2019). Astrocytes play a crucial role in pathogenic progression at the acute stages of ICH (Chiu et al., 2017). Astrocytes activity and astrocyte-derived factors are responsible for both BBB destruction and repairment after brain injury (Michinaga and Koyama, 2019). Following ICH, astrocyte-derived matrix metalloproteinases (MMPs) have been reported to aggravate BBB breakdown and brain damage with possible regulation on TJs (Min et al., 2015; Keep et al., 2018). We found that compared with the bEnd3 monoculture group, co-culture with sham-astrocytes didn’t alter the expressions of ZO-1 and claudin-5 in bEnd3 cells, whereas co-culture with ICH-induced astrocytes and vehicle led to a significant decrease in the expressions of ZO-1 and claudin-5 in bEnd3 cells. To investigate the potential factors that may alter the BBB permeability following ICH, we further evaluated the MMP-9 levels in the co-culture medium by ELISA. We found that MMP-9 expression was significantly increased in the ICH group (Supplementary Figure 1) and did not show many alterations under CYC treatment. The multiple factors secreted in the co-culture medium eventually affect the expression of TJs protein and permeability of BBB. Other mechanisms that may affect the BBB integrity in the bEnd3 astrocytes co-culture system still needs experiments in our further study. Also, we found that compared with the co-culture with ICH-induced astrocytes and vehicle, CYC treatment caused a remarkable decrease in the expressions of ZO-1 and claudin-5 in bEnd3 cells, indicating a protective role of astrocytic SHH signaling pathway on ICH by modulating TJs expression.

The SHH pathway is a critical signaling pathway in a variety of diseases, such as asthma, gastrointestinal system, preeclampsia, bladder cancer, and brain injury (Wang et al., 2008, 2019; Amankulor et al., 2009; Sims et al., 2009; Syed et al., 2016; Hu et al., 2017; Qi et al., 2018; Liao et al., 2019). The potential biological mechanisms of the SHH signaling pathway in CNS may be associated with a wide range of pathological processes, such as neurogenesis, anti-oxidation, autophagy, and anti-apoptosis. Several types of research demonstrated that the SHH signaling pathway may be involved in the integrity of BBB and the endogenous anti-inflammatory response to CNS immune attacks (Alvarez et al., 2011; Singh et al., 2016; Zhen et al., 2018). We appreciate these thoughtful comments. Li et al. (2013) reported the role of the SHH signaling pathway in the experimental SAH model, demonstrating that the SHH signaling pathway could be involved in the pathogenesis of SAH, suggesting possible protective effects on the SAH-induced oxidative stress by the PI3K/Akt pathway. Different from the SAH, which mainly affect the subarachnoid space, ICH showed a marked increase in BBB hyperpermeability, brain edema, and neurological deficit after bleeding into the brain parenchyma (Smith and Eskey, 2011). Thus, the possible contribution of the SHH signaling pathway on ICH has been elucidated in our study, clarifying the protective effect of the astrocytic SHH signaling pathway on ICH by TJs regulation. Our study is the first to show that the inhibition of the SHH signaling pathway by CYC significantly aggravates ICH-induced neurological dysfunction, brain edema, hematoma volume, and BBB permeability in mice. Gli-1 is a crucial regulator of BBB integrity and TJs expressions. As an SMO antagonist, CYC can selectively bind to SMO, ultimately lead to the repression of Gli-1 transcription factors into the nucleus, resulting in lower TJs expressions in ECs throughout the ICH-induced damages *in vivo* and *in vitro*. The potential mechanism may involve the regulation of TJs expressions in ECs through the SHH-Gli-1 axis, as shown in Figure 7. Our study demonstrates that SHH might be used as a target to protect BBB integrity for early treatment in ICH.

SHH signaling pathway has been considered as vital for BBB function *via* TJs regulation. In our study, the SHH-Gli axis was shown to be essential for BBB integrity following ICH, since inhibition of SHH led to severer BBB damage. However, it has been reported that SHH-Ptch also contributes to upregulate vascular endothelial grow factor (VEGF), angiopoietins Ang-1 and Ang-2, which participate in the development of vasculature ingrowth and BBB function (Pola et al., 2001). Thus, SHH may play a protective role in BBB integrity also by promoting VEGF, Ang-1, and Ang-2 production. Thus, the SMO-Gli-1-TJ relation in the protective role of astrocytic SHH in BBB integrity still needs further investigation. In our study, further experiments also are needed to clarify the protective role of astrocyte-derived SHH in ICH (i.e., a transgenic mouse line in which the SHH is overexpressed or knocked down in astrocytes). Furthermore, the agonist of the SHH signaling pathway may be used to identify the potential therapeutic property of the SHH signaling pathway in ICH.

## Conclusion

Our results show that the astrocytic SHH signaling pathway plays a pivotal role in the protection of BBB integrity and function in ICH, suggesting the potential therapeutic value of SHH in ameliorating ICH injury. The underlying mechanism of astrocytic SHH-targeting therapy needs to be further studied.

## Data Availability Statement

All datasets presented in this study are included in the article/Supplementary Material.

## Ethics Statement

All experiments were approved by the Animal Experiments Ethical Committee of Tianjin Medical University General Hospital and Jining Medical University (the number of the approval of ethics statement: 2019-FY-024). This study was managed following the National Institutes of Health Guide for the Care and Use of Laboratory Animals in China.

## Author Contributions

WJ and NZ formulated the concept and designed the study. GX, TZ, and DL performed the studies. XZ, HL, and XL analyzed the data and interpreted the results. PC contributed new reagents or analytic tools. GX, TZ, and ML wrote and revised the article. All authors contributed to the article and approved the submitted version.

## Conflict of Interest

The authors declare that the research was conducted in the absence of any commercial or financial relationships that could be construed as a potential conflict of interest.

## Footnotes

^1^http://rsb.info.nih.gov/ij/

## Abbreviations

ICH: intracerebral hemorrhage
BBB: blood-brain barrier
SHH: sonic hedgehog
TJs: tight junctions
ECs: endothelial cells
CYC: cyclopamine
Ptch-1: patched-1
SMO: smoothened
Gli-1: glioma-associated oncogene homolog-1
HPBCD: hydroxypropyl-β-cyclodextrin
CMC-NA: carboxymethylcellulose sodium
PBS: phosphate-buffered saline
mNSS: modified Neurological Severity Score
GFAP: glial fibrillary acidic protein
EB: Evans blue
GAPDH: glyceraldehyde 3-phosphate dehydrogenase
PHE: perihematomal.
