# Supplementary material for: Astrocytic Sonic Hedgehog Alleviates Intracerebral Hemorrhagic Brain Injury via Modulation of Blood-Brain Barrier Integrity
Source: Front Cell Neurosci. 2020 Dec 3;14:575690. doi: 10.3389/fncel.2020.575690 (PMC7747855; doi:10.3389/fncel.2020.575690)
Supplement: Supplementary file 1 [file Table_1.docx]

**SUPPLEMENTAL MATERIAL**

**Supplementary Table 1**. Modified Neurological Severity Score (mNSS)

| Points |
| --- |
| Motor tests  Raising mouse by the tail 3  1 Flexion of forelimb  1 Flexion of hindlimb  1 Head move > 10° to vertical axis within 30 s  Placing mouse on the floor (normal=0; maximum=3) 3   1. Normal 2. Inability to walk straight 3. Circling toward the paretic side 4. Fall down to the paretic side   Sensory tests 2   1. Placing test (visual and tactile test) 2. Proprioceptive test (deep sensation, pushing the paw against the table edge   To stimulate limb muscles)  Beam balance tests (normal=0; maximum=6) 6   1. Balances with steady posture 2. Grasps side of beam 3. Hugs the beam and one limb falls down from the beam 4. Hugs the beam and two limbs fall down from the beam, or spins on beam   (> 60 s)   1. Attempts to balance on the beam but falls off (> 40 s) 2. Attempts to balance on the beam but falls off (> 20 s) 3. Falls off: No attempt to balance or hang on to the beam (< 20 s)   Reflexes absent and abnormal movements 4   1. Pinna reflex (head shake when touching the auditory the cornea with cotton)   1 Corneal reflex (eye blink when lightly touching the cornea with cotton)  1 Startle reflex (motor response to a brief noise from snapping a clipboard  paper)   1. Seizures, myoclonus, myodystony   Maximum points 18 |

**Supplementary Table 1 for Referees**. **Modified Neurological Severity Score (mNSS)**. One point was given if mice failed to complete a task or lacked a tested reflex; 13 to 18 indicates severe impairment; 7 to 12 indicates moderate impairment; 1 to 6 indicates mild impairment.


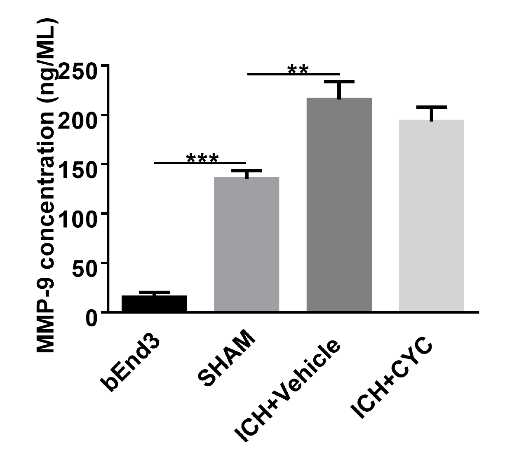


**Supplementary Figure 1**. **Evaluated the MMP-9 levels in the co-culture medium by ELISA *in vitro.*** ICH was induced in mice by injection of autologous blood. The expressions of MMP-9 were measured in bEnd3 cells supernatants that were cultured alone, co-cultured with sham-induced astrocytes, co-cultured with ICH-induced astrocytes and vehicle, and co-cultured with ICH-induced astrocytes and CYC. Compared with the bEnd3 group, MMP-9 expressions were significantly increased in SHAM group and ICH+Vehicle group. Compared with the ICH+Vehicle group, no obvious alteration was showed under CYC treatment. n=3 mice per group. Data are presented as mean ± SEM. **P<0.01, ***P<0.001.


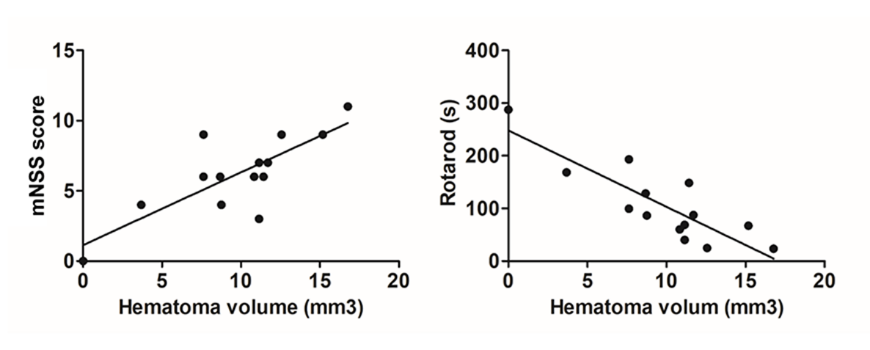


**Supplementary Figure 2**. **Hematoma sizes showed to be correlated with the behavioral scores in CYC-treated mice.** Severer neurological deficits tend to be found in mice with larger hematoma sizes.


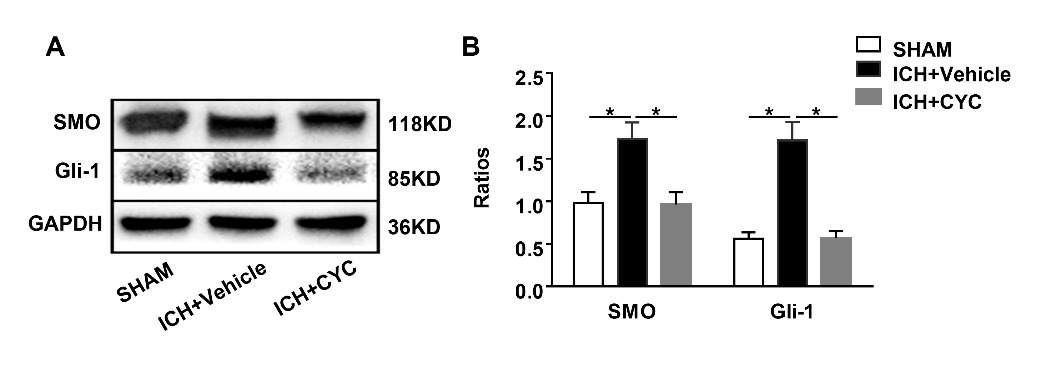


**Supplementary Figure 3**. **CYC inhibits the activation of SHH-Gli-1 axis *in vitro*.** The expressions of SMO and Gli-1in bEnd3 that were co-cultured with sham-induced astrocytes, co-cultured with ICH-induced astrocytes and vehicle, and co-cultured with ICH-induced astrocytes and CYC. Western blot images showed the expressions of SMO and Gli-1in ICH+Vehicle groups were increased as compared to the SHAM group, indicating the SHH signaling pathway activation induced by ICH *in vitro*. The expressions of SMO and Gli-1 in ICH+CYC group were decreased compared to ICH+Vehicle group. n=3 mice per group. Data are presented as mean ± SEM. *P<0.05.
